# Environmentally Efficient 316L Stainless Steel Feedstocks for Powder Injection Molding

**DOI:** 10.3390/polym12061296

**Published:** 2020-06-05

**Authors:** Berenika Hausnerova, Martin Novak

**Affiliations:** 1 Department of Production Engineering, Faculty of Technology, Tomas Bata University in Zlin, Vavreckova 275, 760 01 Zlin, Czech Republic; m8_novak@utb.cz; 2 Centre of Polymer Systems, University Institute, Tomas Bata University in Zlin, nam. T.G. Masaryka 5555, 760 01 Zlin, Czech Republic

**Keywords:** powder injection molding, processability, feedstock, wax binder, 316L stainless steel powder

## Abstract

In this study, environmentally convenient highly metal powder filled feedstocks intended for powder injection molding is presented. The composition of 60 vol % 316L stainless steel gas atomized powder feedstocks containing semicrystalline waxes: acrawax or carnauba wax and paraffin wax, combined with polyethylene glycol and modifier, was optimized to provide defect-free parts. Rheological as well as thermogravimetric analyses supported with scanning electron microscopy and metallography were employed to set up optimum conditions for molding, debinding and sintering. The performance of the novel feedstock was compared with currently available polyolefines-based materials, and results showed an efficiency enhancement due to the substantially lower (about 100 °C) mixing and molding temperatures as well as a reduction of debinding and sintering times at the simultaneous achievement of better mechanical properties in terms of elongation and tensile strength, in comparison to the mass production feedstock.

## 1. Introduction

Injection molding of metal and ceramic powder feedstocks (PIM—Powder Injection Molding) provides high performance products without residual waste. Currently, the interests in PIM are supported by a possibility to directly adopt feedstocks developed for PIM in additive manufacturing and merge these technologies together. Not only inherent compromises between speed of the processes, sintered densities, amount of residual stresses and mechanical integrity [1,2,3], but also demands on binder systems used, are limiting the efficiency of both processing routes. Another aspect is the requirement to make the production less energy consuming, and to employ environmentally benign materials.

Primary (backbone) binders for highly filled metal and ceramic powder feedstocks are mostly based on polyolefins (low- and high-density polyethylenes and polypropylene) [4,5,6]. For ceramic powders, water-soluble binders based on polyethylene glycol (PEG) steadily gain attention [7,8,9,10]. Lower molecular weight of PEG is usually recommended for PIM applications [11,12,13,14]. Sintered parts made from feedstocks containing up to 70 vol % of PEG in the binder were reported to have no cracks when the solvent was debound [13,14]. As a surfactant, stearic acid (SA) is typically used for both metal and ceramic feedstocks [4,5,6].

Recently, we showed that semicrystalline waxes such as carnauba wax (CW) and acrawax (AW) have a promising potential as binders for high powder loadings [7,9], and they could substitute the role of polyolefin backbone in PIM binder compositions [10]. According to the FTIR, calorimetry and contact angles analyses [7,15], AW have high values of polar component of surface energy and proved to have twice as strong interactions to PEG than CW. On the other hand, based on the contact angles analysis [7], CW may overtake the role of SA as a surfactant. Furthermore, PEG can be easily debound in water, and CW is a renewable resource (*Copernicia prunifera palm*).

The aim of this research is to optimize processing conditions for highly filled 316L stainless steel compounds based on PEG, acrawax or carnauba wax, paraffin wax and stearic acid in order to achieve improved performance of 316L PIM parts, and simultaneously more efficient processing.

## 2. Materials and Methods

Binders consisted of 59 wt % polyethylene glycol varying in molecular weight (PEG 4000 and PEG 6000, SINOPOL, Sino-Japan Chemical Co., Ltd.; Taipei, Taiwan), 28 wt % acrawax (AW, ethylene bis stearamide, Acrawax C, atomized; LONZA, Basel, Switzerland) or carnauba wax (CW, 2442, Kahl GmbH & Co. KG, Trittau, Germany), 12 wt % paraffin wax (PW, paraffinum solidum, FAGRON, Olomouc, Czech Republic) and 1 wt % stearic acid (SA, P-LAB a.s., Prague, Czech Republic). The particular ratios of the components in the binder compositions arise from our previous research [10]. As a metal powder, 316L stainless steel powder having particle size at 90%—16 μm, pycnometric density 7.76 g/cm^3^ and tap density 4.6 g/cm^3^ (Osprey 316L, SANDVIK OSPREY, Sandviken, Sweden) was used.

Auxiliary chemicals for performed analyses were: corrosion inhibitor (Inhibitor 4000, 2 vol %; Zschimmer & Schwarz GmbH & Co. KG, Lahnstein, Germany) used during water debinding, and aqueous solution of HCl+HNO_3_+FeCl_3_ employed as an etchant for microstructure observation.

Transition temperatures were obtained from differential scanning calorimetry (DSC, Perkin Elmer DSC 7 apparatus, Perkin Elmer, Inc., Waltham, MA, USA) in the temperature range from 20 to 180 °C (the second heating scan, 10 °C/min, nitrogen atmosphere, standard aluminum pan, 10 mg sample). Homogeneity of feedstocks was controlled with a scanning electron microscope (SEM, Phenom Pro, Thermo Fisher Scientific, Phenom-World B.V., Eindhoven, The Netherlands), while an optical microscope (Olympus GX5, Olympus IMS, Tokyo, Japan) was used to perform microstructure analyses of carefully polished and etched sintered samples. Rheological properties of feedstocks were determined using a capillary rheometer (RG 50, Göttfert Werkstoff-Prüfmaschinen GmbH, Buchen, Germany) on a capillary having a length/diameter ratio of 20/1 in the apparent shear rate range (10–4000) s^−1^.

Thermogravimetric analysis (TGA) was performed on the samples (TGA Q50, TA Instruments, New Castle, DE, USA); the samples (20.0 ± 0.1) mg were prepared from injection molded samples after water debinding. For analysis of the binder systems, the samples were taken from the pellets after mixing. Nitrogen (balance 40 mL/min; sample 60 mL/min) was selected as an atmosphere during TGA to achieve an inert atmosphere and simulate conditions of thermal debinding from 30 to 700 °C with a speed of 5 °C/min.

Sintered density was obtained through a water immersion method. Vickers (HV) microhardness was measured on a Micro-Combi Tester instrument (CSM Instruments SA, Peseux, Switzerland) on 4 different places at each of 3 samples for each investigated speed (5, 10 and 15 °C/min), i.e., 12 measurements for each speed. Elongation at break, tensile and yield strength were measured with a tensile testing machine (ZWICK Materialprüfung 1456, ZwickRoell GmbH & Co. KG, Ulm, Germany) according to ASTM standard method E8M-00.

## 3. Results and Discussion

The compositions of feedstocks reflect our previous research devoted to the quantification of interactions among various polymers used as binder components [9,15]. First, the binders were prepared by mixing components in an agitator, and then fed through the hopper to the extruder. The extruded binder was then granulated on a grinding machine, mixed with the steel powder and extruded, granulated and extruded again to guarantee the homogeneity of the feedstocks. The loading of 60 vol % of steel powder was chosen as a maximum due to a relative fragility, which samples exhibited after water debinding, when most of the PEG was removed from the green parts.

Mixing was performed on a counter-rotating twin-screw extruder (Brabender Plasti-corder PL 2000, Brabender GmbH & Co., Duisburg, Germany). Based on a DSC analysis revealing the transition temperatures of binder components, the starting compounding temperatures were 160 °C and 90 °C for AW and CW based binders, respectively. During optimization, they were lowered in 10 °C steps for the AW binder, and by smaller steps (5 °C) for the CW binder until reaching acceptable mixing with temperature profiles finally adjusted in 1 °C steps as (65/60/45) °C with the mixing rate of 100 rpm, and (55/58/45) °C at the 60 rpm for AW and CW based binders, respectively.

**Figure 1:**
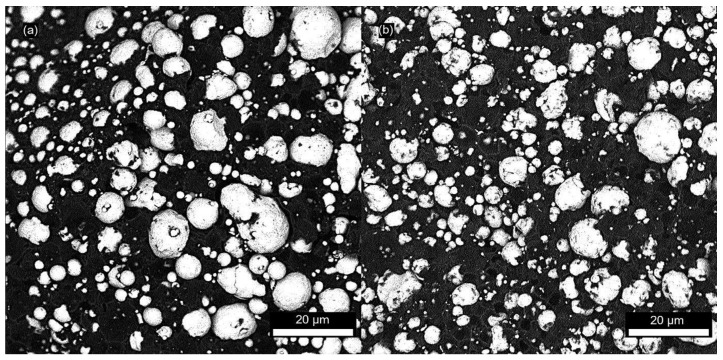
SEM micrograph of: (**a**) acrawax (AW) based feedstock; (**b**) carnauba wax (CW) based feedstock.

Then, the powder was admixed into the binder, and homogeneity of the obtained feedstocks was observed with SEM (Figure 1). The prepared feedstocks were observed in BSD (Backscatter electron detector) full mode that provides high contrast micrographs of the powder and binder. As can be seen, the individual particles of the spherical shape are evenly distributed within the binder, and no aggregates are observed.

During the compounding of the AW feedstock, the temperature in the middle zone raised up 15 °C due to the heat generated by friction (even though the screw speed was kept at only 20 rpm). In the case of the CW binder, the friction-heat-generated temperature increase was negligible. The final temperature profiles were (65/60/55) °C for the compound containing AW and (60/60/50) °C for CW based feedstock.

It should be noted that the optimized mixing temperatures are about 100 °C lower than those recommended for currently available materials [16]. For both feedstocks, they were below the transition temperatures of waxes (CW: 82.7 and 77 °C, AW: 144.5 and 65.4 °C). Vuluga et al. [17] also obtained the best dispersion of their halloysite nanotubes in a AW/poly(methyl methacrylate) matrix well below the melting temperature of AW.

Further, the feedstocks were molded into rectangular shapes (118 × 12 × 4 mm) as well as tensile test samples according to MPIF Standard 50 [18]. It was carried out on a PIM injection molding machine (Allrounder 370S, Arburg, ARBURG GmbH + Co. KG, Lößburg, Germany) with the injection unit having a 100 mm stroke, screw diameter 20 mm and L/D 20; the crew is hardened with the geometry suitable for the processing of highly abrasive feedstocks. Injection molding parameters and the temperature profile for injection molding were optimized to the values depicted in Table 1.

**Figure 2:**
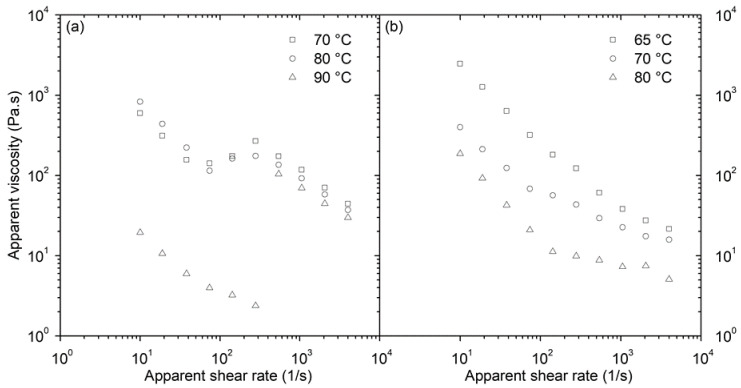
Apparent viscosity data of: (**a**) AW-based feedstock; (**b**) CW-based feedstock.

The apparent viscosity of the AW based feedstock at 70 and 80 °C shows overshot at certain shear rates (Figure 2a). At lower shear rates (up to 100 1/s) the cause of viscosity is pseudoplastic, suggesting particle or binder molecule orientation and ordering with flow. Upon further increase of shear rate, particles cannot form layers and slide over each other (first reported by Hoffman [19]), and shear thinning turns into a dilatant flow. There is still considerable uncertainty about the source of such behavior. The mechanism proposed by Barnes [20] is that with increasing shear rate the layers formed in the pseudoplastic flow region become disrupted, and flow turns into dilatant, when they are fully eliminated. Thus, every highly concentrated compound exhibits dilatant flow if proper flow conditions are selected. Such structure reorganization has been determined for this type of feedstock also for temperatures in the range of 150 to 170 °C, i.e., above the melting point of AW [21]. Only at 90 °C, there is apparently a processing window with the pseudoplastic cause of the flow, which might be connected to the transition temperature region, where AW converts from the beta to the alpha form [17], influencing the dispersion balance of the powder within the binder.

In the case of carnauba wax feedstocks (Figure 2b) the change in the slope of viscosity as a function of shear rate can be distinguished as the result of apparent yield stress, which arises from the three-dimensional structure formed by the particles within this binder.

PIM compounds are typically materials lacking the symmetry in their flow cause, and therefore, we have proposed the eight parameter model to describe their viscosity [22], and most recently, master curves, which might sufficiently intercept the flow performance of various PIM feedstocks with a complex dilatant/pseudoplastic flow behavior [23].

**Figure 3:**
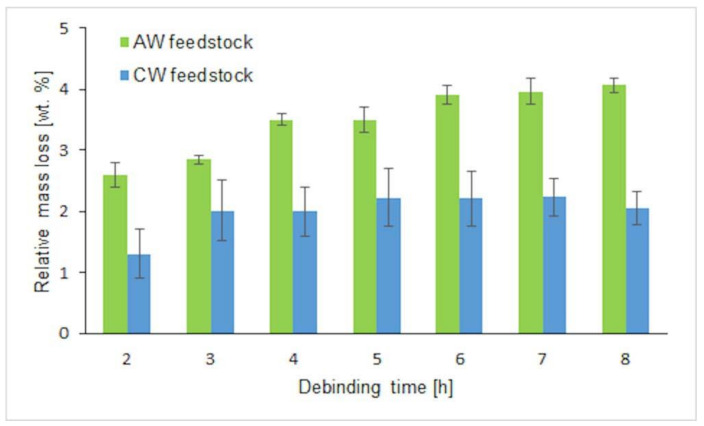
Relative mass loss during water debinding of CW- and AW-based feedstocks.

**Figure 4:**
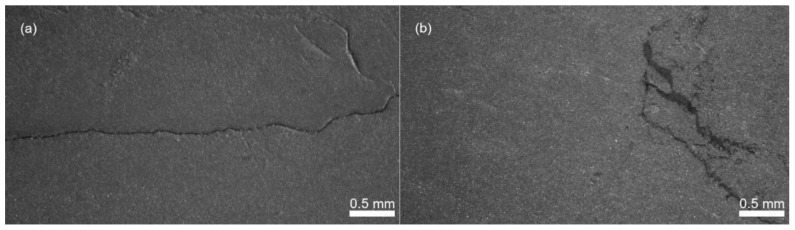
Distortions and cracks of CW-based feedstocks during water debinding: (**a**) 2 h; (**b**) 15 h.

Debinding of the molded samples in the demineralized water containing the corrosion inhibitor allowed the creation of pore channels through the components. A water debinding temperature of 50 °C was chosen below the lowest transition temperature of the binder components. The water debinding time was optimized based on the measurement of the relative weight losses; 4 mm thick samples were taken out of the debinding bath in hour intervals, dried for 48 h at 50 °C in an oven, and weighed (Figure 3). The optimum debinding time for AW based feedstock was determined to be 7 h with the relative loss of the mass (4 ± 0.1 wt %) representing the loss of the PEG component of 80.5 wt %. After 8 h, the samples started to exhibit a surface erosion and cracking. For CW based feedstock, the surface delamination had been already visible after 2 h of the PEG removal causing the broad scatter of the data, and after 15 h the weight loss was still less than 3 wt % (60.5% of PEG) accompanied with severe crack formations (Figure 4).

**Figure 5:**
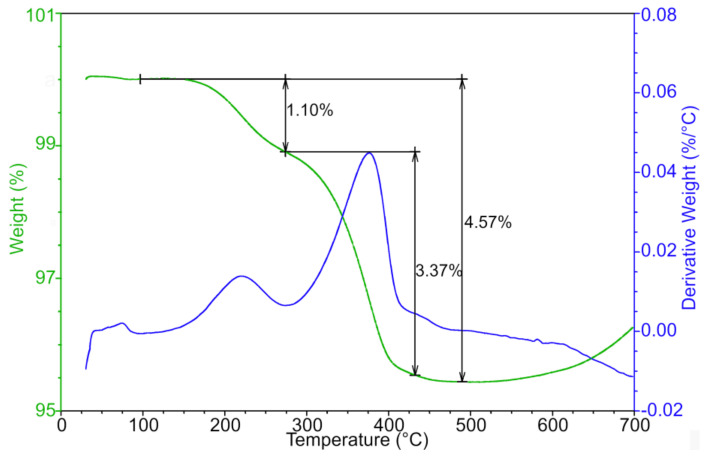
Thermogravimetric analysis of AW based feedstock after water debinding.

Thermal debinding and sintering was performed in a sintering furnace (CLASIC CZ s.r.o., Revnice, Czech Republic). The conditions were set up according to the results of the TGA analysis (Figure 5). In general, more gradual decrease in weight represents a lesser chance for crack formation and allows for higher thermal debinding/sintering speeds. The first loss of 0.9 wt % is attributed to PW and SA, which have been reported to appear between 140 and 275 °C (PW) [24,25], and from 150 to 300 °C (SA) [26,27]. The second loss of 2.05 wt % means mainly removal of AW [17,28], and the last weight drop of 0.96 wt % is linked to the evaporation of the rest of the AW and remains of the PEG (both PEG 4000 and PEG 6000 have been reported to start to degrade between 350 °C and 430 °C [29]). This three-step process is gradual, which is desirable for shape retention and prevention of cracks.

**Figure 6:**
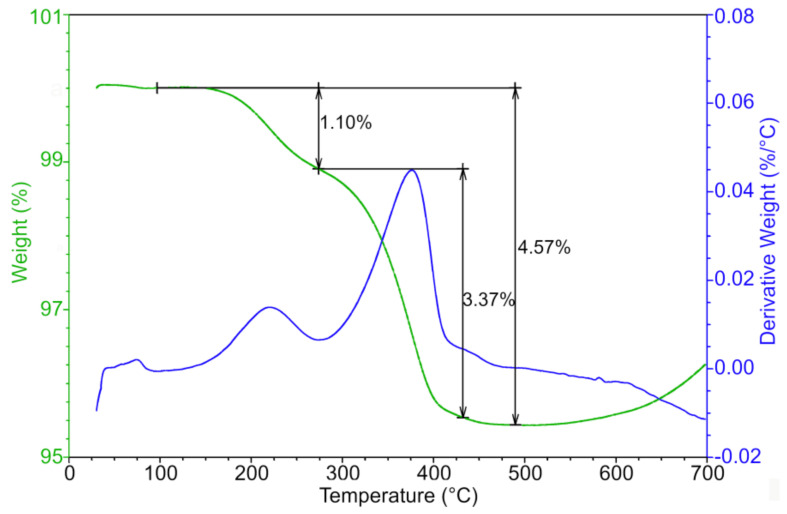
Thermogravimetric analysis of CW-based feedstock after water debinding.

Although the weight loss of the CW based feedstock (removal of CW component reported between 350 and 500 °C [30,31]) is rather similar (Figure 6) to that of the AW based sample, the previous failure of a withdrawal of PEG by water immersion caused cracks of the debound samples.

**Figure 7:**
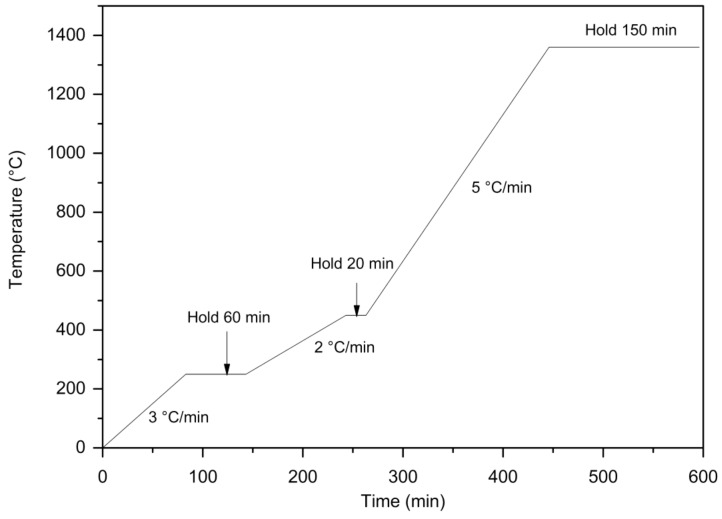
Thermal debinding and sintering profile optimized for AW-based feedstock (300 mbar, nitrogen).

As the thermal debinding/sintering atmosphere, nitrogen has been chosen for 316L stainless steel. The reason to employ nitrogen is in accordance with the aim to produce cost-efficient PIM parts (it is approximately five times cheaper than hydrogen). According to the previous studies [32], it provides samples of the highest strength, but leaves the part brittle with the ductility of only 15%. Mechanical properties of sintered samples depend also on temperature and length of sintering. Higher temperatures may lead to a formation of larger grains and adversely affect the tensile strength of a sample [5]. The AW based samples were debound and sintered at various conditions (Figure 7): first hold temperatures of 180, 250 and 270 °C, second hold temperature of 450 °C, hold times of 10, 20, 30 and 60 min and speeds of 2, 3 and 5 °C/min. From 450 to 1360 °C, the influence of the sintering speed (speeds of 5, 10 and 15 °C) on the mechanical properties of the samples was investigated.

The first hold temperature of 250 °C proved to be adequate, whereas 180 °C caused significant distortions in the samples, and 270 °C allowed for the formation of the severe cracks. The speed in the interval from 250 to 450 °C is necessary to be kept at 2 °C only to allow the smooth thermal debinding of the remaining binder components. Concerning tested hold times in this region (10, 20, 30 and 60 min), all times shorter than 60 min caused cracks and blistering on the surface. On the other hand, the second hold time of 20 min at the temperature of 450 °C appears to be sufficient.

The optimized thermal debinding and sintering speed can be seen in Figure 7; it runs in a nitrogen atmosphere with a pressure of 300 mbar from a room temperature up to 250 °C with the speed 3 °C/min and holds 60 min, then the speed is lowered to 2 °C/min from 250 °C to 450 °C and holds 20 min, where the rest of the binder is thermally removed, and finally, the sintering is carried out with the speed of 5 °C/min from 450 up to 1360 °C, where this sintering temperature is held for 150 min. This shortens the sintering stage of the process to 600 min even when using the slowest speed of 5 °C/min (504 min for 10 °C/min, 474 min for 15 °C/min). In the case of polyolefin binders, the overall sintering times for 316L reported are 900 min for (PW, PEG-600, SA and low density polyethylene (LDPE)) binder [33], 1000 min for (PW, LDPE, SA) binder [34], or 630 min for commercially available feedstock polyMIM^®^ 316L D 222 E.

Table 2 summarizes mechanical properties of the AW based sintered parts obtained for various sintering speeds in the range of temperatures (450–1360) °C. The speed of 5 °C/min provided the highest tensile strength of ≥520 MPa in comparison to ≥450 MPa for the mass production feedstock polyMIM^®^ 316L D 222 E. The yield stresses achieved are also substantially higher than that of the commercial feedstock (≥140 MPa).

Vickers microhardness generally correlates with yield strength, but it should be mentioned that the microhardness of the sintered PIM parts was measured on relatively porous testing samples, which influences the results. Overall, lower elongation values can be attributed to the sintering medium (nitrogen) as already mentioned above. Standard deviations of elongation are usually not provided for PIM sintered parts, e.g., [4,32], however they might provide important additional information about the number of defects in the final sintered structure. The variation in the elongation obtained for the speed 5 °C/min (26.7%) is similar to that obtained for additive manufactured 316L stainless steel parts by Natali et al. [35].

**Figure 8:**
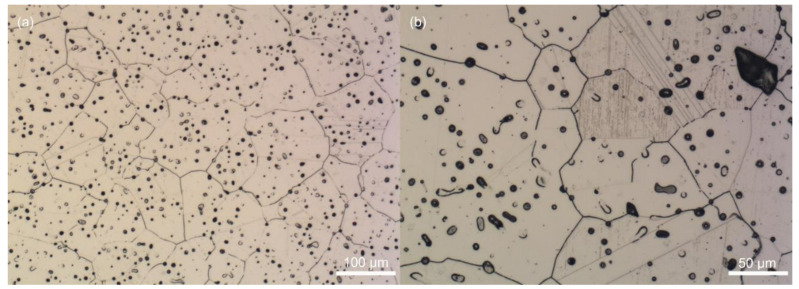
Microstructure of 316L stainless steel sintered at 5 °C/min (**a**) with the detail of deformation-induced martensite (**b**).

The mechanical properties can be explained through the differences in the relative densities and microstructure. The sintered density value (Table 2) obtained for the speed of 5 °C/min corresponds to the relative density 93.7% of the pycnometric density. Generally, a structure of 316L steel is austenitic [2,3,32,36], however, martensite transformation may occur as a deformation-induced transformation. The behavior of austenitic stainless steel during deformation is dependent on the value of stacking fault energy (SFE) and temperature of the deformation. 316L grade steels have relatively high values of the SFE, and therefore, the amount of transformed martensite and twining is assumed to be low, however, as shown, for example, by Sulima et al. [37], it can occur. As can be seen in Figure 8, the pores for the sintering speed of 5 °C/min were relatively numerous, but small. The microstructure is austenitic with rare deformation twins, and even deformation–induced martensite.

**Figure 9:**
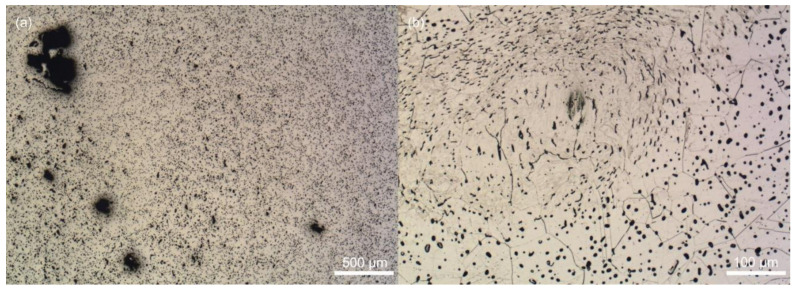
Microstructures of 316L stainless steel: (**a**) large defects on sintered area for 10 °C/min speed; (**b**) vortexes located around larger pores at 15 °C/min speed.

When the sintering speed was raised to 10 °C/min, a number of defects occurred (Figure 9a) explaining lower tensile strength as well as sintered density of this series of the samples. The heating rate of 15 °C/min resulted in the structure corresponding to the relative density value between those of the samples sintered at 5 and 10 °C/min. However, defects in terms of vortexes located around larger pores as shown in Figure 9b were detected for this sintering profile. Deformation-induced martensites are located mainly in the vicinity of the vortexes and the borders of the defects.

Overall, the biggest grain size of austenite (90 ± 35 μm) was found for the samples sintered at 5 °C/min speed, while the size of the grains for 10 and 15 °C sintering speeds were (53 ± 25 μm) and (62 ± 29 μm) respectively. Thus, it is assumed that the heating rates faster than 5 °C/min induce a stress into samples, which prevents a growth of grains, and a proper closure of pores. While in general, decrease in a grain size would lead to a higher tensile strength [5], in the case of studied materials, densification and pores size/shape drive the final properties instead of the grain size. A decrease in porosity generally leads to the increase of yield strength, which is, on the other hand, suppressed by the grain growth occurring simultaneously [38]. Thus, it was also found that it may remain constant [38] similarly to the AW-based samples tested.

## 4. Conclusions

Stainless steel 316L feedstocks, containing acrawax or carnauba wax as the binder component, were proceeded via powder injection molding (PIM). The feedstocks were mixed and injection molded at temperatures about 100 °C lower than the traditional polyolefin-based PIM compounds. Debinding was performed via combined solvent (immersion in water) and thermal routes. While in the case of acrawax-based feedstocks the immersion of the green parts was obtained after 7 h, the carnauba wax-based samples resulted in cracks and delamination. The thermal debinding and sintering profile optimized for acrawax-based feedstock is shorter than those of currently available PIM feedstocks, even for the slowest sintering speed tested (5 °C/min). Mechanical properties in terms of tensile (≥520 MPa) and yield (≥258 MPa) strengths improved in comparison to ≥450 MPa and ≥140 MPa, respectively, of the mass production feedstock. The efficiency enhancement of the processing of 316L stainless steel in terms of energy (at least 300 kWh of energy per one sintering cycle for the laboratory-scale sintering oven), time, and consumption of chemicals and media (hydrogen substituted with nitrogen) was achieved.

## Figures and Tables

**Table 1:** Injection molding conditions for AW- and CW-based feedstocks.

Injection Molding Parameter	Material	
	AW Feedstock	CW Feedstock
Temperature—nozzle [°C]	75	65
Temperature—zone 1 [°C]	95	70
Temperature—zone 2 [°C]	85	90
Temperature—zone 3 [°C]	80	80
Temperature—zone 4 [°C]	75	60
Temperature—zone 5 [°C]	20	30
Screw stroke [mm]	60	70
Cooling time [s]	30	10
Injection pressure [bar]	1000	500
Hold pressure/time 1 [bar]/[s]	800/5	400/5
Hold pressure/time 2 [bar]/[s]	150/2	50/0.5

**Table 2:** The effect of sintering speed on mechanical properties of AW based sintered parts.

Mechanical Property	Speed [°C/min]		
	5	10	15
Tensile strength [MPa]	557 ± 34	535 ± 45	546 ± 41
Yield strength [MPa]	264 ± 6	267 ± 5	264 ± 2
Elongation [%]	30 ± 8	25 ± 11	27 ± 9
Sintered density [g/cm^3^]	7.27	7.16	7.22
Vickers Microhardness [HV]	150 ± 6	145 ± 7	145 ± 11
